# Transgenic Tobacco Overexpressing Tea cDNA Encoding Dihydroflavonol 4-Reductase and Anthocyanidin Reductase Induces Early Flowering and Provides Biotic Stress Tolerance

**DOI:** 10.1371/journal.pone.0065535

**Published:** 2013-06-18

**Authors:** Vinay Kumar, Gireesh Nadda, Sanjay Kumar, Sudesh Kumar Yadav

**Affiliations:** 1 Biotechnology Division, CSIR-Institute of Himalayan Bioresource Technology, Council of Scientific and Industrial Research, Palampur, Himachal Pradesh, India; 2 HATS Division, CSIR-Institute of Himalayan Bioresource Technology, Council of Scientific and Industrial Research, Palampur, Himachal Pradesh, India; Cinvestav, Mexico

## Abstract

Flavan-3-ols contribute significantly to flavonoid content of tea (*Camellia sinensis* L.). Dihydroflavonol 4-reductase (DFR) and anthocyanidin reductase (ANR) are known to be key regulatory enzymes of flavan-3-ols biosynthesis. In this study, we have generated the transgenic tobacco overexpressing individually tea cDNA *CsDFR* and *CsANR* encoding for DFR and ANR to evaluate their influence on developmental and protective abilities of plant against biotic stress. The transgenic lines of *CsDFR* and *CsANR* produced early flowering and better seed yield. Both types of transgenic tobacco showed higher content of flavonoids than control. Flavan-3-ols such as catechin, epicatechin and epicatechingallate were found to be increased in transgenic lines. The free radical scavenging activity of *CsDFR* and *CsANR* transgenic lines was improved. Oxidative stress was observed to induce lesser cell death in transgenic lines compared to control tobacco plants. Transgenic tobacco overexpressing *CsDFR* and *CsANR* also showed resistance against infestation by a tobacco leaf cutworm *Spodoptera litura*. Results suggested that the overexpression of *CsDFR* and *CsANR* cDNA in tobacco has improved flavonoids content and antioxidant potential. These attributes in transgenic tobacco have ultimately improved their growth and development, and biotic stress tolerance.

## Introduction

Flavonoids comprise one of the largest groups of plant secondary metabolite. These are widespread throughout the plant kingdom and are found to be accumulated in different organs and tissues of plants. They are mainly involved in providing protection to plants against predation, pathogen (bacteria and fungi) attack, and act as effective repellant and prevent feeding by herbivores [1]. They are the major quality factors for forage crops. The higher concentration of flavonoids can decrease the palatability of forage crops. In some forage crops, the presence of flavonoids can also be a positive trait and recognized as health beneficial compounds to the ruminant animals by reducing pasture bloat [2]–[5]. Several different classes of flavonoids including anthocyanins, flavonols, isoflavones, monomeric flavan-3-ols (catechins and epicatechins) and oligomeric flavan-3-ols (proanthocyanidins; PAs) contribute to the growth and survival of plants under UV exposure as well as against pathogen and herbivores [4]. At the same time, they impart astringency and flavor to beverages such as wine, fruit juice and tea [6].

**Figure 1:**
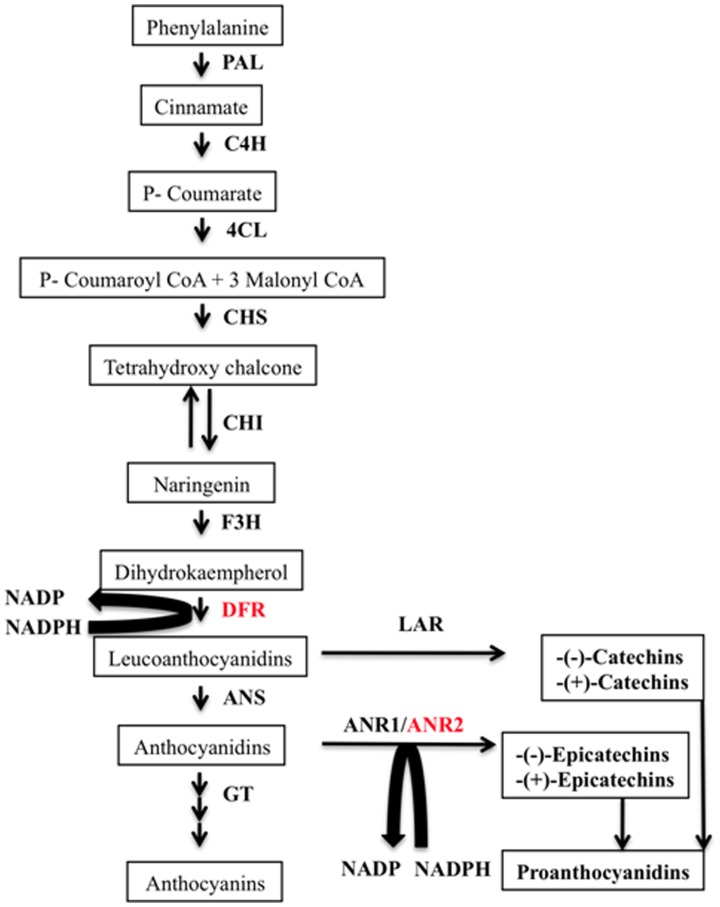
General outline of anthocyanins and flavan-3-ols biosynthetic pathway in plants. The enzymes are: PAL, Phenylalanine ammonia-lyase; C4H, cinnamate 4-hydroxylase; 4CL, 4-coumaroyl CoA-ligase; CHS, chalcone synthase; CHI, chalcone isomerase; F3H flavanone-3-hydroxylase; DFR, dihydroflavonol 4-reductase; LAR, leucoanthocyanidin reductase; ANS, anthocyanin synthase; ANR1, anthocyanin reductase1; ANR2, anthocyanin reductase2; GT, Glucosyl transferase.

The genetics and biochemistry of flavonoid biosynthetic pathway has been extensively studied in number of plant species. The broad outline of flavonoid biosynthesis pathway in plants is shown in Figure 1. Flavan-3-ols biosynthesis shares anthocyanidin biosynthesis pathway from phenylalanine to leucoanthocyanidin (flavan-3, 4-diol). In this pathway, DFR (dihydroflavonol 4-reductase; EC 1.1.1.219) catalyzes the production of leucoanthocyanidin. Leucoanthocyanidin is a common substrate for the production of both flavan-3-ols and anthocyanins, and is converted to anthocyanidin by anthocyanidin synthase (ANS; EC 1.14.11.19) and to flavan-3-ols (catechin) by leucoanthocyanidin reductase (LAR; EC 1.17.1.3). Anthocyanidin is converted to epicatechin by anthocyanidin reductase (ANR; EC 1.3.1.77) [2], [3]. The DFR and ANR have been considered aspivotal enzymes of flavan-3-ols biosynthesis. They belong to the short chain dehydrogenase/reductase or DFR super family. By sequence similarity, both are closely related to each other [7]. Genes encoding these two enzymes are characterized by a similar exon/intron pattern and enzymatic proteins contained an amino acid sequence motif for NADPH binding. The DFR has been identified and characterized from several plant species [8]–[11]. The transgenic tobacco overexpressing DFR encoding cDNA from *Medicago truncatula* (*MtDFR*) has been produced and studied with respect to flavonoids content [12]. Also, the transgenic rice overexpressing *MtDFR* has been reported for altered metabolites profile [13]. Similarly, ANR has also been identified and characterized from several plant species [14]–[18]. Transgenic tobacco overexpressing *MtANR* has been generated and analyzed for its effect on PAs content [19]. The PAs accumulation was also reported to be altered by suppressions of *ANR1* and *ANR2* in *Glycine max* [20]. Recently, the proteins encoded by *ANR1* and *ANR2* genes from leaf tissue of a blister blight-resistant tea cultivar TRI2043 have been documented for different level of epimerase activity and exhibited similar kinetic properties [21].

Flavan-3-ols are the building block of PAs. They are also reported for their antioxidant potential and free radical scavenging activity [5], [6]. Tea (*Camellia sinensis*) plant contains an extraordinarily high ∼25–30% of flavan-3-ols on leaf dry weight basis. Recently, progress has been made towards elucidation of flavonoid biosynthesis in tea. A cDNA encoding for DFR (Flavan-3-ol NADPH-dihydromycertin reductase; EC 1.1.1.219) has been cloned from tea cultivar UPASI 10. *CsDFR* is 1,413 bp full-length cDNA with ORF of 1,044 bp (115–1158) and encoding a protein of 347 amino acid residues [10]. Also, a cDNA encoding for anthocyanidin reductase (ANR: Flavan-3-ol: NAD(P)^+^ oxidoreductase: EC 1.3.1.77) has been cloned from tea cultivar UPASI 10. *CsANR* is 1,233 bp full-length cDNA with ORF of 1,014 bp (79–1092) and encoding a protein of 337 amino acid residues. The *CsANR* cDNA from tea cultivar UPASI 10 showed 97% and 80% homology at nucleotide level with previously reported *CsANR2* (Accession no. GU992400) and *CsANR1* (Accession no. GU992402) from blister blight-resistant tea cultivar TRI2043, respectively. Hence, this has suggested that *CsANR* used in this study is *CsANR2*. Interestingly, CsANR activity has been found to be directly correlated with epicatechin content in tea [17].

Previously, a number of transgenic plants have been developed and characterized with respect to engineer flavonoid biosynthetic pathway [15], [20], [22]–[26]. Since tea is reported to contain very high content of flavonoids particularly flavan-3-ols, the genes encoding enzymes of regulatory steps of flavonoid pathway from tea were used in this study to engineer tobacco. Recent studies have described that *CsF3H*, *CsANR* and *CsDFR* encoding enzymes are crucial for flavonoid biosynthesis in tea [10], [17], [27]. Among them, CsANR and CsDFR belong to DFR super family. In view of all this, present study was planned to generate transgenic tobacco overexpressing tea cDNA *CsDFR* and *CsANR* encoding for DFR and ANR. To see the influence of *CsDFR* and *CsANR* cDNA overexpression, transgenic tobaccos were analyzed for flavonoids content and antioxidant potential. They were further assessed for the influence of these transgenes on their development and protective abilities against biotic stress.

## Materials and Methods

### Preparation of pCAMBIA-*CsDFR* and pCAMBIA-*CsANR* construct, tobacco transformation and transgenic confirmation

The cDNA sequence of *CsDFR* and *CsANR* is available at NCBI with Accession number AY64027 and AY641729, respectively. The isolated cDNAs of *CsDFR* and *CsANR* from *C. sinensis* cultivar UPASI-10 were cloned into pCAMBIA 1302 plasmid between *Nco I* and *Bgl II* restriction sites. This was resulted in the formation of recombinant construct pCAMBIA-*CsDFR* and pCAMBIA-*CsANR*. These constructs were transferred into *Agrobacterium tumefaciens* strain LBA4404 by triparental mating. *A. tumefaciens* harboring pCAMBIA-*CsDFR* and pCAMBIA-*CsANR* constructs were used for leaf disc transformation of tobacco (*Nicotiana tabacum* cv *Xanthi* nc) following the standard transformation protocol [28]. Plants rooted on hygromycin selection (50 mg/ml) were screened for the presence of *CsDFR* and *CsANR* transgene by carrying out PCR with genomic DNA and gene specific primers (*CsDFR* forward primer 5′-ATGGAAGCCCAACCGACAGCTC-3′ and reverse primer 5′-TCAATT CTTCAAAATCCCCTTAGCCT -3′; *CsANR* forward primer 5′-ATGAAAGACTCTGTTGCTTCTGCC-3′ and reverse primer 5′-TTA AACCTTGTTGCCATTGACAGG-3′). The reaction conditions were as follow: 94°C for 1 min, and then 35 cycles of 30 s at 94°C, 30 s at 54°C (for *CsDFR*) and 58°C (for *CsANR*), and 2 min at 72°C. PCR products were separated by agarose gel electrophoresis and visualized with ethidium bromide. T_1_ transformants were self-pollinated and the seeds obtained from T_1_ were analyzed for segregation by germinating on half strength Murashige and Skoog medium supplemented with hygromycin (50 mg/ml). One month old plants were transferred in green house in pots containing the manure, sand and soil in 1∶1∶2 ratios. Tobacco plant transformed with pCAMBIA vector having no transgene *CsDFR* or *CsANR* was used as control (mock). The third leaf of three plants of each independent transgenic line as well as control tobacco plant was harvested and used for various sample preparation.

### RNA isolation and semiquantitative PCR

The 100 mg of leaf tissue of transgenic lines as well as control tobacco plants was ground in liquid nitrogen and total RNA was isolated using RNeasy Plant Mini Kit (Qiagen, Germany). The total RNA from each sample was treated with DNase I to remove DNA contamination. cDNA was prepared according to manufacturer's protocol (Invitrogen, USA) using 2 µg of total RNA, 250 ng oligo dT_12–18_, 200 U of superscript III RT, and 10 mM dNTPs in a 20 µl reaction volume. Equal quantity of cDNA was used as template in PCR with gene-specific primer sets for probing the expression of *NtPAL*, *NtC4H*, *Nt4CL*, *NtCHS*, *NtCHI*, *NtF3H*, *NtFLS*, *NtDFR, NtANR1, NtANR2* and *NtANS* genes. All primer sequences used in this study are shown (Table S1). The primers of *NtANR1* and *NtANR2* were designed in such a way that RT-PCRs could be performed specifically. Linearity between the amount of input RNA and the final PCR products was verified and confirmed. After standardizing the optimal amplification at exponential phase, PCR was carried out with the conditions of 94°C for 4 min for 1 cycle, 94°C for 30 s, 52°C (*NtANS*), 54°C (*NtC4H*, *Nt4CL*, *NtCHS, NtANR1, NtANR2*), 58°C (*NtDFR*, *NtPAL, NtCHI, NtF3H* and *NtFLS*) for 30 s, and 72°C for 30 s for 27 cycles. The amplified products were separated on 1% agarose gel and visualized with ethidium bromide staining. A gel documentation system (Alpha DigiDoc™, Alpha Innotech, USA) was used to scan the gel and changes in the gene expression were analyzed by calculating integrated density values (IDV) using AD-1000 software. The 26S rRNA-based gene primers were used as internal control for relative gene expression studies [29].

### Measurement of morphological and yield parameters

The parameters namely days to flowering, capsules number, seed yield and thousand seed weight were analyzed. These parameters were analyzed in eight plants of each selected lines of *CsDFR* and *CsANR* overexpressing tobaccos as well as control tobacco plants. The capsules were counted at their maturation time and seed yield and weight of thousand seeds was measured after harvesting the capsules of *CsDFR* and *CsANR* overexpressing transgenic lines as well as control tobacco plants.

### Estimation of flavonoids content

The 250 mg of freeze-dried leaf powder of transgenic lines as well as control tobacco plants was taken for extract preparation. Total flavonoids content in the extract was determined using the previously described method [30]. Total flavonoids content was expressed as mg/g quercetin equivalent. Flavan-3-ols such as catechin (Cat), epicatechin (EC) and epigallocatechin (EGC) were analyzed by HPLC method [31]. Briefly, 1 g of tobacco leaf tissue of each transgenic tobacco line in triplicates was freeze dried, and used for flavan-3-ols extraction with 70% methanol. Flavan-3-ols were measured by Merck Hitachi HPLC (Darmstadt Germany) using C18 Licrocart column (250 mm×5 mm×5 µm) and absorbance was read at 210 nm. The flavan-3-ols (+)-Cat, (−)- EC and (−)-EGC were used as standard from Sigma for estimation of respective constituent.

### Estimation of DPPH radical activity

Antioxidant activity of the methanolic extract of leaves of three plants from each transgenic as well as control tobacco plants was performed. The ability of plant extract to oxidize stable DPPH (diphenylpicryl-hydrazyl) free radicals was estimated as described earlier [32]. The initial absorbance of DPPH in methanol was measured using spectrophotometer at 517 nm until the absorbance remained constant. A total of 50 µl of extract was added to 1950 µl of 0.1 mM methanolic DPPH solution. The mixture was incubated at room temperature for 30 min before the change in absorbance at 517 nm was observed. The percent of inhibition was calculated using the formula,
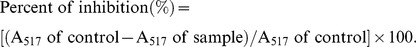



### Evans blue staining for H_2_O_2_-induced cell death

Fresh leaves from three plants of each transgenic line as well as control tobacco plants were treated with 5 mM H_2_O_2_ for 2 h as described previously [33]. Samples were then incubated with 0.05% Evans blue for 15 min, followed by extensive washing to remove unbound dye. Dye bound to dead cells was solubilized with 50% methanol and 1% sodium dodecyl sulfate at 50°C for 30 min and quantified by taking absorbance at 600 nm.

### Leaf disc non-choice test

To check the possible anti-feeding behavior of tobacco cutworms (*Spodoptera litura*), leaf discs of 6 cm diameter were prepared from transgenic lines as well as control tobacco plants and experiment was performed with some modification as described earlier [34]. These discs were kept in different petri dishes containing wet filter paper with 10 larvae (less than 24 h old) of *S. litura* and allowed to feed for 7 consecutive days. Experiment was performed in three replicates for each transgenic line. The petri dishes were kept at 27±2°C, 60±5% RH and a photoperiod of 16 h: 8 h (Light: Dark). The growth behavior of cutworm caterpillar was represented as percentage growth inhibition and calculated as
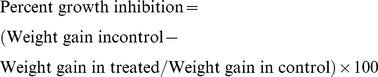



### Phytohormone analysis

Endogenous gibberellin (GA_3_) and auxin in the form of free indole-3-acetic acid (IAA) content of control as well as transgenic tobacco lines were determined by HPLC method as described earlier [35]. Five gram fresh tissue of control as well as transgenic tobacco lines were homogenized with 70% (v/v) methanol and stirred overnight at 4°C. The extracts were used for further extraction with ethyl acetate and diethyl ether stepwise. After that, samples dissolved in methanol were used for HPLC analysis and absorbance was read at 210 nm for GA_3_ and 265 nm for IAA. The GA_3_ and IAA from Sigma were used as standard for estimation of respective constituent.

### Protein estimation

Total protein content in various extracts was estimated following the Bradford method [36]. Standard was prepared with BSA and used for protein estimation.

## Results

### Confirmation of transgenic tobacco overexpressing *CsDFR* and *CsANR* cDNA

**Figure 2:**
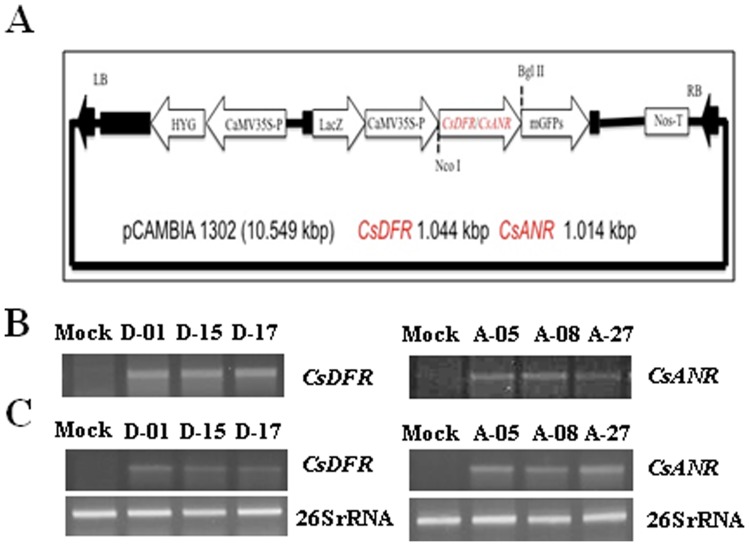
Generation of *CsDFR* and *CsANR* transgenic tobacco. A, Graphic representation of pCAMBIA 1302 vector with cDNA of *CsDFR* and *CsANR*. *CsDFR* and *CsANR cDNA* was inserted in-between the *NcoI* and *BglII* restriction site of pCAMBIA 1302. B, Genomic DNA PCR confirmed the insertion of *CsDFR* and *CsANR cDNA* in plant genome of transgenic lines. C, Semi-quantitative PCR documented the transcript expression levels of *CsDFR and CsANR* in transgenic tobacco lines. Housekeeping gene 26S rRNA was used as internal control for expression study and experiments were repeated at least three times with similar results.

Transgenic tobaccos were generated using Agrobacterium-mediated transformation of recombinant construct pCAMBIA-*CsDFR* and pCAMBIA-*CsANR* (Fig. 2A). The integration of *CsDFR* and *CsANR* cDNA in the genome of transgenic lines was confirmed through PCR. In PCR, transgene specific primers were used with total genomic DNA extracted from the mature leaves and PCR products were confirmed by sequencing. A representative picture depicting the integration of *CsDFR* and *CsANR* cDNA is shown only for three lines of each transgenic tobacco (Fig. 2B). A specific band of 1.044 and 1.014 kbp has indicated the introduction of *CsDFR* and *CsANR* cDNA respectively in the transformed tobacco lines. T2 homozygous lines of these transgenic tobaccos were selected and confirmed three lines were further used for *CsDFR* and *CsANR* expression analysis.

For *CsDFR* and *CsANR* transcript expression analysis, total RNA was isolated from leaves of confirmed transgenic lines and used forcDNA synthesis. A primer set specific to *CsDFR* and *CsANR* cDNA was used for PCR amplification. Detection of *CsDFR* and *CsANR* transcripts in transgenic lines has suggested the constitutive expression of transgenes. While in control tobacco plants, no expression was observed for the transgenes (Fig. 2C). Three *CsDFR* overexpressing transgenic lines as D-01, D-15 and D-17 and three *CsANR* overexpressing transgenic lines as A-05, A-08 and A-27 showed relatively better expression of *CsDFR* and *CsANR* transcripts, respectively. Therefore, these lines were used for various analyses.

### Tobacco plants overexpressing *CsDFR* and *CsANR* showed early flowering and significantly higher yield

**Figure 3:**
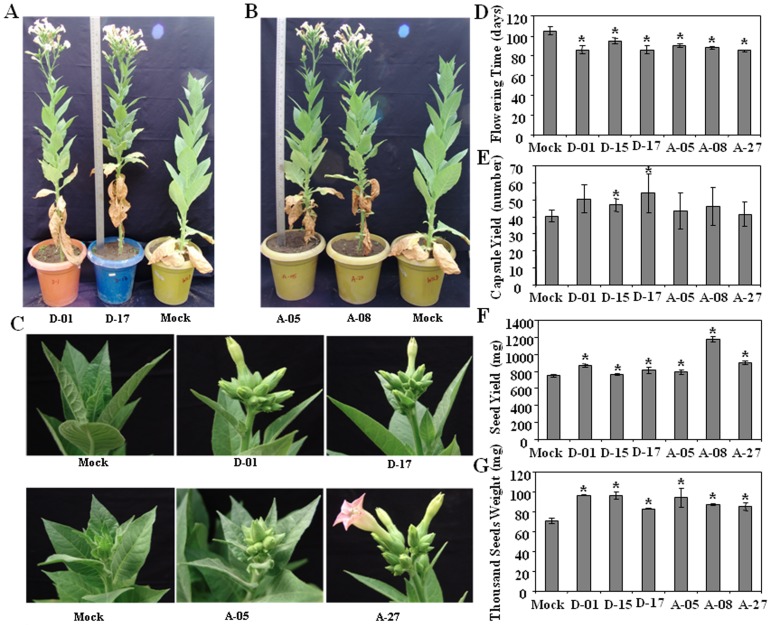
Morphological characterization and yield parameters of *CsDFR* and *CsANR* overexpressing transgenic lines vis-à-vis mock tobacco plants. *CsDFR* and *CsANR* overexpressing transgenic lines were longer than mock tobacco plants (A, B). Close up views showing early flowering in *CsDFR* and *CsANR* transgenic lines compared to mock tobacco (C). The *CsDFR* and *CsANR* overexpressing transgenic lines produced early flowering as compared to control tobacco plants (D). The capsule yield (E), seed yield (F) and seed weight per thousand seed (G) in *CsDFR* and *CsANR* overexpressing transgenic lines as compared to mock tobacco plants. Mean ± SD from three replications are shown. Statistical significance is indicated as (*) for P<0.05.

Transgenic lines vis-à-vis control plants were morphologically characterized for flowering time. The relative growth of transgenic lines overexpressing *CsDFR* and *CsANR* compared with the control is shown in Fig. 3A, B. The *CsDFR* and *CsANR* overexpressing transgenic lines were flowered early and completed their life cycle 10–15 days in advance compared to control. Close up views showing the phenotype of early flowering in *CsDFR* and *CsANR* overexpressing transgenic tobacco lines (Fig. 3C). Transgenic tobacco lines overexpressing *CsDFR* (D) and *CsANR* (A) flowered after an average of 86 days (D-01), 95 days (D-15), 86 days (D-17), 90 days (A-05), 88 days (A-08), and 85 (A-27) days of seed germination compared to 105 days to flower after seed germination in control tobacco plants (Fig. 3D). The number of capsules and seed yield per plant was improved in *CsDFR* and *CsANR* transgenic lines as compared to control tobacco plants (Fig. 3D). The capsule yield was not uniformly improved. However, there was an uniform improvement in seed yield per capsule of transgenic lines as compared to control tobacco plants (Fig. 3 E, F). The weight of thousand seeds in transgenic lines was found to be higher than control tobacco plants (Fig. 3G).

### Overexpression of *CsDFR* and *CsANR* increased *NtCHS, NtANR2* transcript expression and flavonoids accumulation in transgenic tobacco

**Figure 4:**
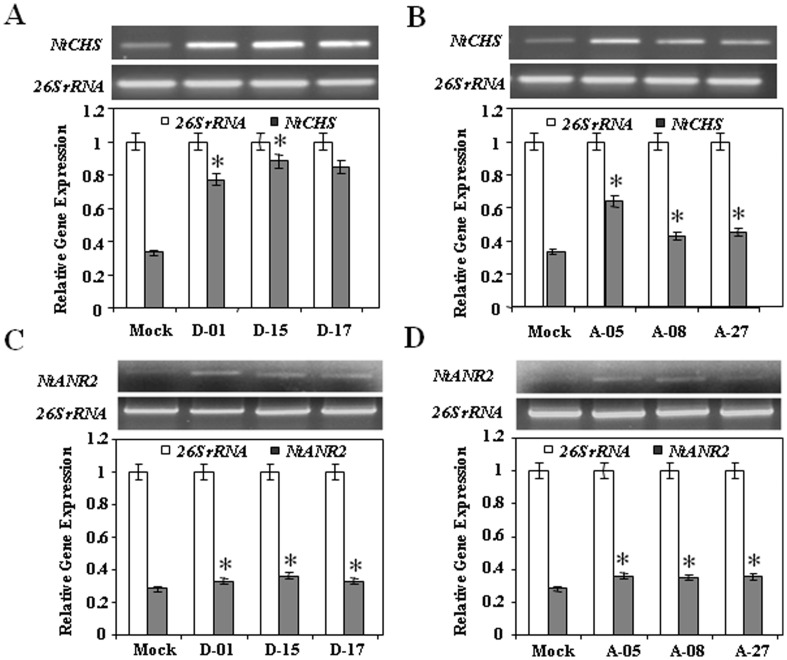
Transcript expression level of *NtCHS* and *NtANR2* gene enhanced in *CsDFR* and *CsANR* transgenic tobacco. A, Transcript expression level of *NtCHS* in *CsDFR* transgenic lines. B, Transcript expression level of *NtCHS* in *CsANR* transgenic lines. C, Transcript expression level of *NtANR2* in *CsDFR* transgenic lines. D, Transcript expression level of *NtANR2* in *CsANR* transgenic lines. Expression of 26S rRNA was used as internal control and experiment was repeated at least three times. Below gel pictures relative level of expression is shown with bar diagram and values are mean of three replications with *error bars* indicating ± SD. Statistical significance is indicated as (*) for P<0.05.

Interestingly, *CsDFR* and *CsANR* overexpressing transgenic lines showed upregulation in transcript expression levels of native tobacco *NtCHS* and *NtANR2* genes (Fig. 4A–D). While there was no significant change in the transcript levels of various other native tobacco *NtPAL*, *NtC4H*, *Nt4CL*, *NtCHI*, *NtF3H*, *NtDFR*, *NtFLS, NtANR1* and *NtANS* genes of flavonoid pathway in both *CsDFR* and *CsANR* overexpressing transgenic lines (Fig. S1).

**Figure 5:**
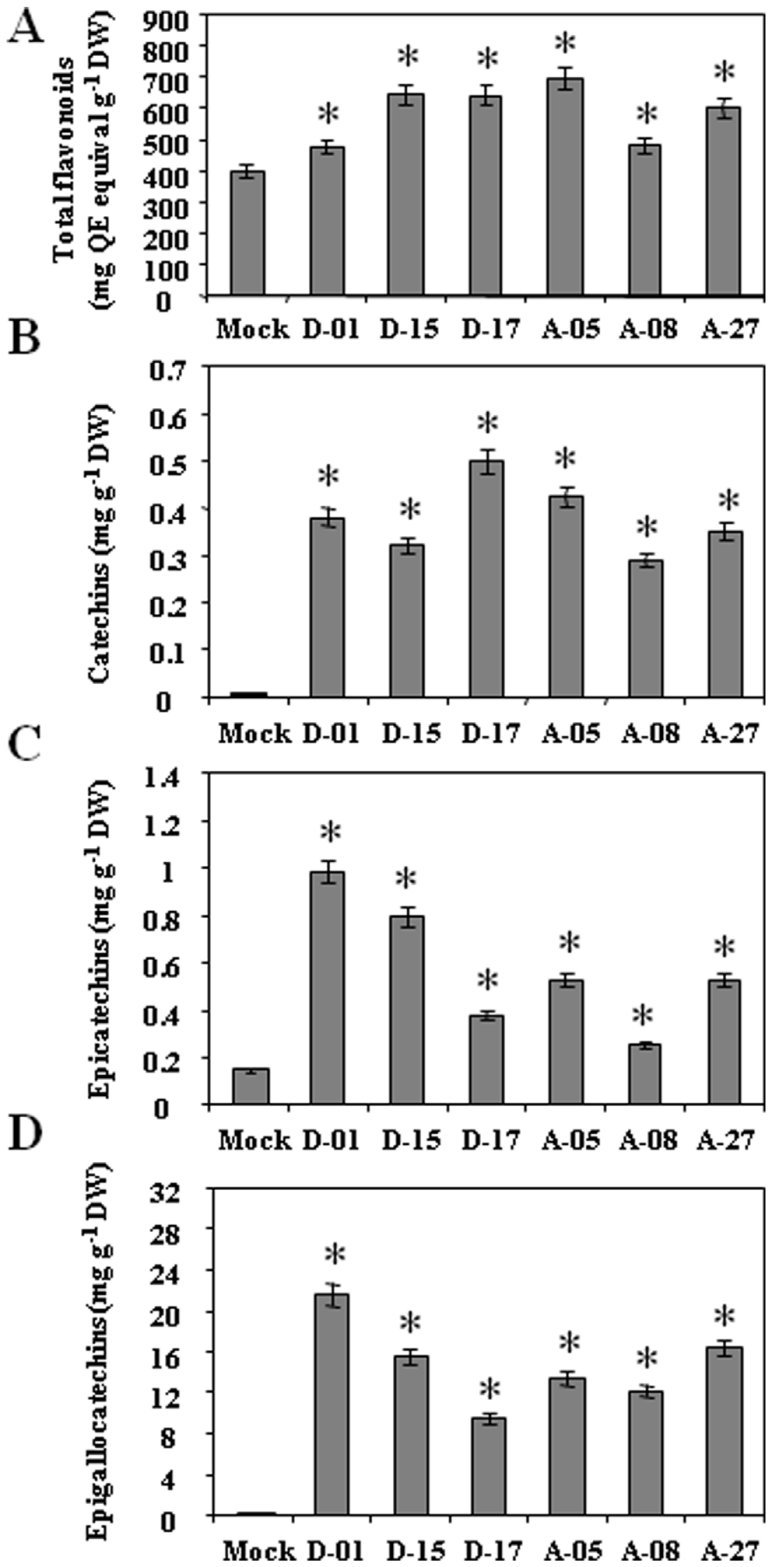
The *CsDFR* and *CsANR* overexpressing transgenic lines show higher contents of total flavonoids and increased flavan-3-ols content in transgenic tobacco compared to control tobaccos. Three flavan-3-ols namely catechin, epicatechin and epigallocatechin were measured in *CsDFR* and *CsANR* transgenic lines vis-à-vis control tobacco. Total flavonoids in *CsDFR* and *CsANR* overexpressing transgenic tobaccos (A). The catechin (B), epicatechin (C) and epigallocatechin (D) contents in *CsDFR* and *CsANR* transgenic lines as well as control tobacco plants. Data is the mean of three replications with *error bars* indicating ± SD. Statistical significance is indicated as (*) for P<0.05.

The relative amount of total flavonoids, and flavan-3-ols (Cat, EC, and EGC) was determined in the transgenic lines vis-à-vis control tobacco plants. Total flavonoids content of control tobacco was 39.8 mg quercetin equivalent (QE) g^−1^ DW. While, the total flavonoids content was estimated higher in the range of 47.63–69.77 mg QE g^−1^ DW in *CsDFR* and *CsANR* overexpressing transgenic lines (Fig. 5A). To check the influence of *CsDFR* and *CsANR* overexpression on flavan-3-ols in tobacco, Cat, EC and EGC content was estimated by HPLC. Only (+)-Cat, (−)- EC and (−)-EGC flavan-3-ols were detected in tobacco. The Cat, EC and EGC were found to be increased in both *CsDFR* and *CsANR* overexpressing transgenic lines. The Cat level was estimated in range of 0.38–0.5 mg g^−1^ DW in *CsDFR* and *CsANR* overexpressing transgenic lines as compared to 0.07 mg g^−1^ DW in control tobacco plants (Fig. 5B). Similarly, EC content was also increased and found in the range of 0.25–0.98 mg g^−1^ DW in *CsDFR* and *CsANR* overexpressing transgenic lines as compared to 0.167 mg g^−1^ DW in control tobacco plants (Fig. 5C). EGC content was observed in range of 9.4–21.5 mg g^−1^ DW in *CsDFR* and *CsANR* overexpressing transgenic lines as compared to 0.22 mg g^−1^ DW of control tobacco plants (Fig. 5D). Spectra of the HPLC runs, indicating identified peaks for Cat, EC and EGC and their respective standards are shown in Figure S2. DMACA staining of young leaf of *CsDFR* and *CsANR* overexpressing transgenic tobacco also showed higher accumulation of flavan-3-ols compared to control tobacco plants (Fig. S3).

### Overexpression of *CsDFR* and *CsANR* enhanced antioxidant potential and provided better resistance to oxidative damage in transgenic tobacco

**Figure 6:**
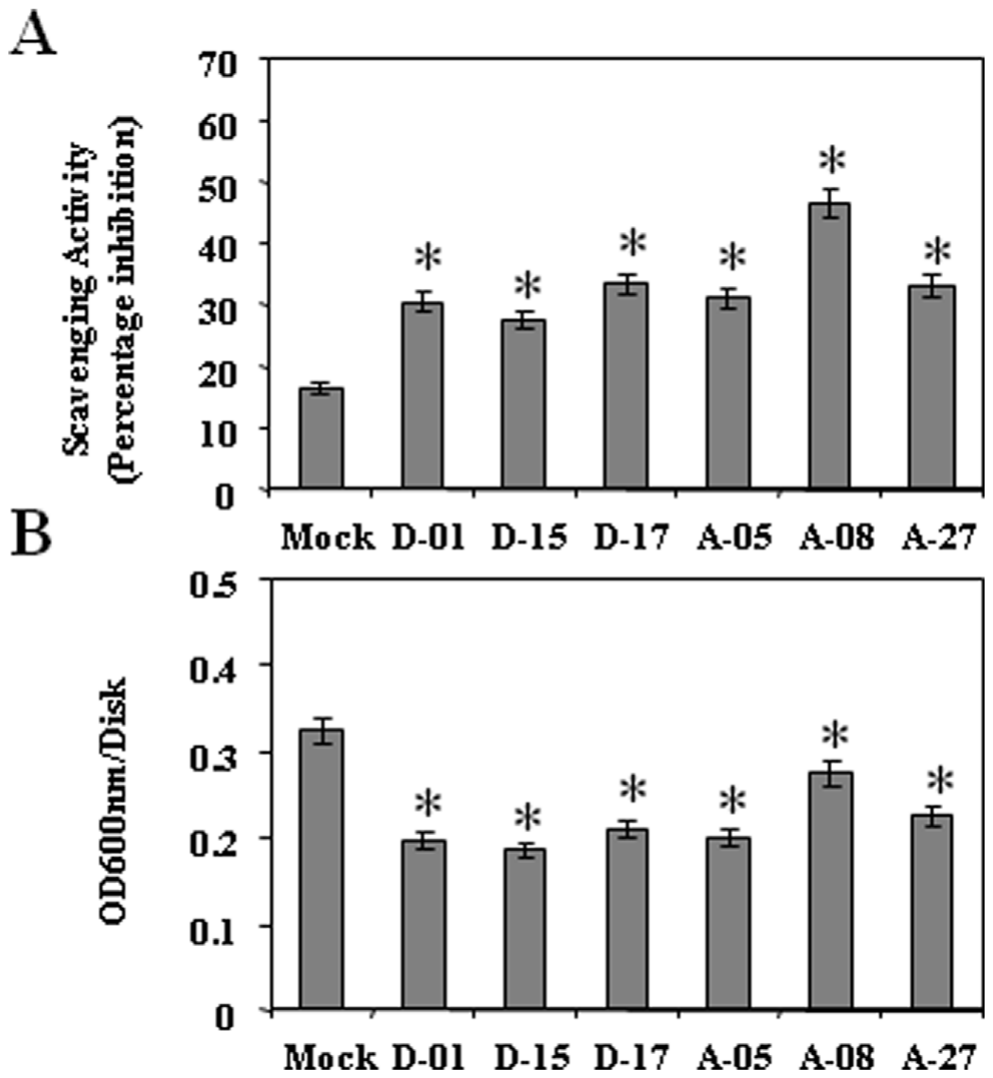
*CsDFR* and *CsANR* overexpression increased free radicals scavenging activity and lowered oxidative stress induced cell death. A, The scavenging activity measured by DPPH method in *CsDFR* and *CsANR* overexpressing transgenic lines. B, H_2_O_2_ induced cell death in *CsDFR* and *CsANR* overexpressing transgenic lines. Data is the mean of three replications with *error bars* indicating ± SD. Statistical significance is indicated as (*) for P<0.05.

To check antioxidant potential, ability of the plant extract of *CsDFR* and *CsANR* overexpressing transgenic lines and control tobacco to scavenge a stable free radical DPPH was measured. Methanolic extract of the leaves of *CsDFR* and *CsANR* overexpressing transgenic lines showed higher free radical scavenging activity for DPPH by 70–185% as compared to control tobacco plants (Fig. 6A). Result suggested the improved antioxidant potential of transgenic tobacco upon *CsDFR* and *CsANR* overexpression.

Since *CsDFR* and *CsANR* overexpressing transgenic lines showed higher levels of flavonoids and enhanced antioxidant potential, the cellular response of transgenic lines to oxidative damage was analyzed by determining ROS-induced cell death. The *CsDFR* and *CsANR* overexpressing transgenic lines showed apparent tolerance to oxidative damage monitored in the form of H_2_O_2_ induced cell death. The H_2_O_2_ induced cell death rate was lesser by 15–43% in *CsDFR* and *CsANR* overexpressing transgenic lines as compared to control tobacco plants.

### Transgenic lines exhibited anti-herbivores effect

**Figure 7:**
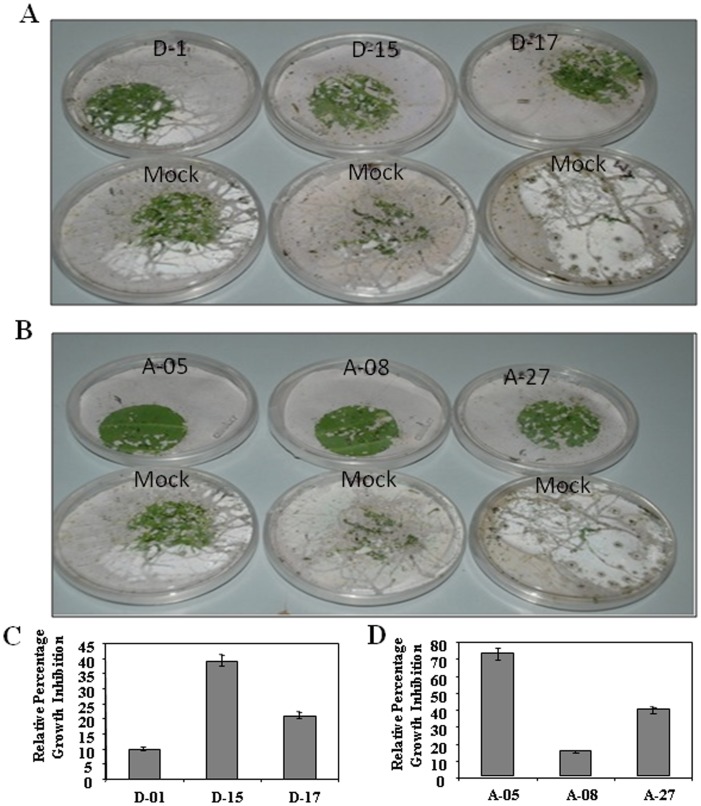
*CsDFR* and *CsANR* overexpression provided anti-herbivores effect against *S. litura* in transgenic tobacco. picture showed less feeding by *S. litura* on leaf discs of *CsDFR* transgenic tobacco line (A) and *CsANR* transgenic tobacco line (B) as compared to leaf discs of control tobacco plants. The relative percentage growth inhibition of *S. litura* feeding on leaf discs of selected *CsDFR* lines (C) and *CsANR* lines (D) as compared to relative percentage growth inhibition on leaf discs of control tobacco plants. Data is the mean of three replications with *error bars* indicating ± SD.

To see the anti-herbivores effect of *CsDFR* and *CsANR* overexpression in tobacco, leaf discs of transgenic lines were exposed to one-day-old *S. litura* larvae. On third day, there was visible difference of feeding by *S. litura* on leaf discs of transgenic tobacco and control tobacco. The leaf discs of *CsDFR* and *CsANR* transgenic lines showed lowest feeding by *S. litura* as compared to control leaf discs (Fig. 7A, B). In other words, leaf discs of *CsDFR* and *CsANR* overexpressing transgenic tobacco showed stronger negative effect on growth and survival of *S. litura* larvae relative to leaf discs of control. This response of transgenics could be due to toxic effects of higher accumulated flavonoids to *S. litura* larvae. Hence, the observed negative effect on the larvae was because they could not eat transgenic leaves and died because of starvation. This was particularly evident in all lines of *CsDFR* and *CsANR* overexpressing transgenic tobacco. Percentage growth inhibition of larvae feeding on leaves of D-01, D-15 and D-17 was 10%, 36% and 20% respectively relative to the growth of larvae feeding on control leaf discs (Fig. 7C). Similar results were observed for *CsANR* overexpressing transgenic lines. Percentage growth inhibition of larvae feeding on leaves of A-05, A-08 and A-27 was 70%, 15% and 40% respectively relative to growth of larvae feeding on control leaf discs (Fig. 7D). The lower survivorship and slower growth of larvae on *CsDFR* and *CsANR* overexpressing transgenic lines has indicated the reduced vigor of *S. litura* larvae.

### Transgenic lines show modulation in endogenous GA_3_ and IAA levels

**Figure 8:**
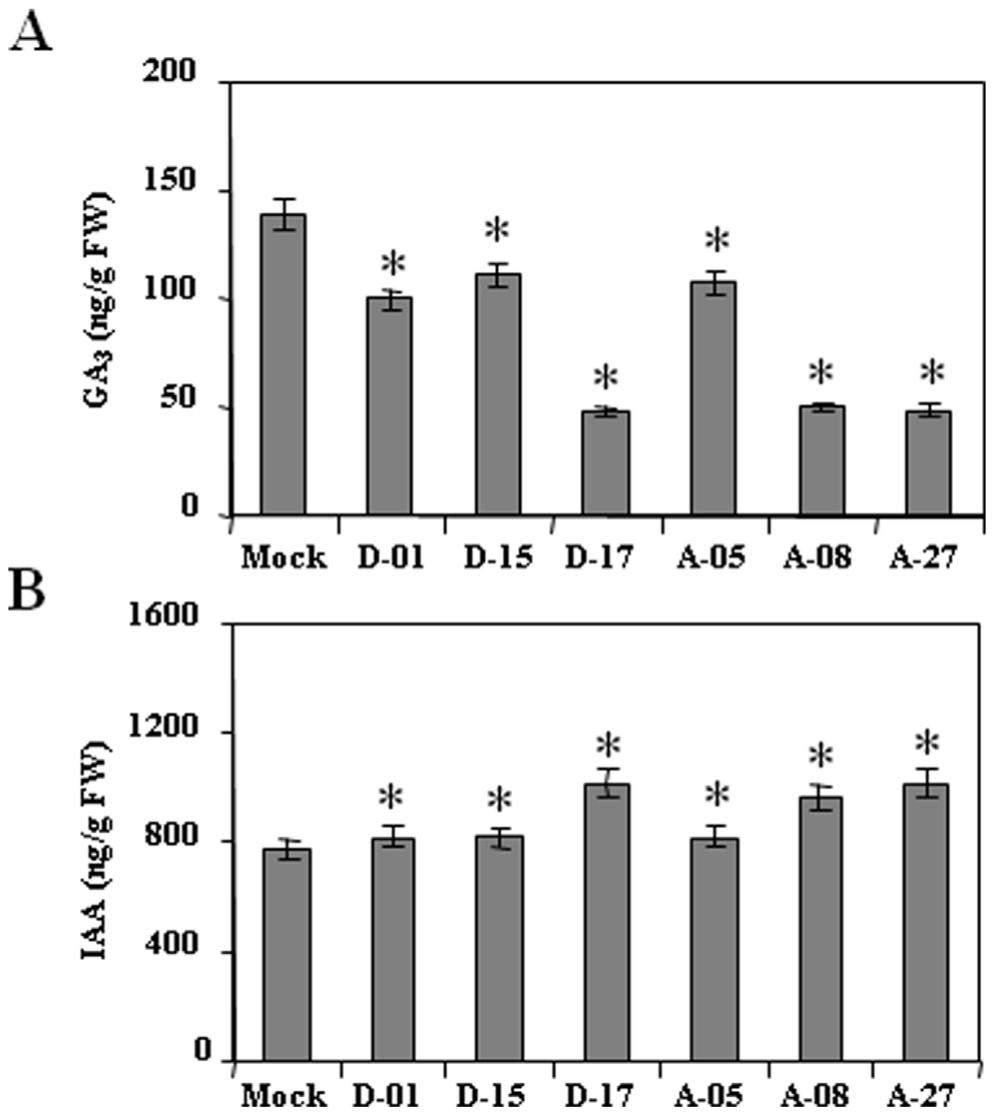
Modulation in phytohormones level in *CsDFR* and *CsANR* overexpressing transgenic lines. Endogenous GA_3_ was decreased (A) and free indole acetic acid (IAA) was increased (B) in transgenics. Data is the mean of three replications with *error bars* indicating ± SD. Statistical significance is indicated as (*) for P<0.05.

To check the influence of *CsDFR* and *CsANR* overexpression on phytohormone levels, the endogenous GA_3_ and IAA levels were measured by HPLC. The GA_3_ level was found to be decreased in transgenic tobacco lines as compared to control tobacco plants. GA_3_ level was observed in the range of 49–110.3 ng/g FW in *CsDFR* and *CsANR* overexpressing transgenic lines compared to 138.80 ng/g FW in control tobacco plants (Fig. 8A). On contrary, the IAA level was found to be higher in transgenic lines as compared to control tobacco plants. IAA level was observed in the range of 810.5–1010.8 ng/g FW in *CsDFR* and *CsANR* overexpressing transgenic lines as compared to 780.2 ng/g FW in control tobacco plants (Fig. 8B).The spectra of HPLC runs with identified peaks for these two phytohormones and standard are shown in Figure S4.

## Discussion

Flavonoids are ubiquitous in their distribution and are important for various fundamental functions in plants. They act as phytoalexins against pathogen and predators and impart astringency to plants making them unpalatable [37], [38]. They also increase antioxidant potential, provide protection against stresses and increase agronomical, economical and medicinal value of several plants [1], [5], [38]. Flaovnoids in the dietary source provide a major source of antioxidants to combat diseases and improvement of human health [3], [5]. Extraordinary high content of flavonoids in tea plant has encouraged us to investigate its flavonoid pathway. *CsF3H*, *CsDFR* and *CsANR* encoding enzymes have been identified as important for flavonoids biosynthesis in tea [10], [17], [27]. Among them, *CsDFR* and *CsANR* encoding enzymes are known to catalyze stereo-specific reduction of substrate using NADPH as a cofactor. Therefore, in this study transgenic tobacco overexpressing cDNA from tea encoding DFR and ANR enzymatic proteins were generated. These transgenic tobaccos were analyzed to see the influence of overexpression of *CsDFR* and *CsANR* transgene onto flavonoids accumulation, developmental and protective abilities against biotic stress. *CsDFR* and *CsANR* overexpressing transgenic tobacco lines have produced 10–15 days early flowering, better capsules and thousand seed weight than control tobacco plants. However, the mutants compromised at different steps in the flavonoid biosynthetic pathway have been reported for affect on shoot/flower number, overall architecture and stature, and thousand seed weight [39]. Similarly, *FLS* silenced transgenic tobaccos were reported with lesser height, delayed flowering and lower yield than control tobacco plant [31]. The present study on overexpression of *CsDFR* and *CsANR* in tobacco has reaffirmed the link between flavonoids and reproductive phenotype by displaying early flowering, improved fruit number, seed yield and thousand seed weight. However, the mechanisms for these changes are not well understood. The possible mechanisms could be either direct interaction of flavonoids with unidentified molecular targets or indirect effects mediated by the ability of flavonoids to modulate the levels of GA and IAA or through ROS regulation [39]. Plant hormones are known to affect the accumulation of secondary metabolites and vice versa [30], [39], [40]. In water deficit condition, the accumulated flavonoids content was observed to decrease endogenous GA_3_ level and increase endogenous IAA level in leaves of *Scutellaria baicalensis* [40]. The decrease in GA_3_ level and increase in endogenous IAA level was also observed in transgenic tobacco overexpressing *CsDFR* and *CsANR*. High level of GA_3_ inhibited the flowering in citrus plants [41]. While topical application of IAA has induced flowering in *Arabidopsis* [42]. Thus, the modulation of endogenous GA_3_ and free IAA level in *CsANR* and *CsDFR* transgenic tobacco lines could be possible reason for early flowering, improved fruit number, seed yield and thousand seed weight.

Altered flavonoids content has been reported through metabolic engineering of flavonoid pathway genes in several transgenic plants [20], [23], [25], [31], [43]. Transgenic tobacco overexpressing *CsDFR* and *CsANR* cDNA encoding for enzymatic proteins showed higher contents of total flavonoids than control tobacco plants. Also monomeric flavan-3-ols such as Cat, EC and EGC were accumulated to higher extent in both *CsDFR* and *CsANR* overexpressing transgenic tobacco than control plant. Importantly, overexpression of *CsDFR* and *CsANR* cDNA in transgenic tobacco has also upregulated the expression of *NtCHS* and *NtANR2* gene, while other genes encoding enzymes of flavonoid pathway remained unaffected. Flavonoids are synthesized in a series of enzymatic steps starting with chalcone synthase (CHS) that catalyzes the condensation of 4-coumaroyl-CoA and malonyl-CoAs. CHS catalyzes the first regulatory step and regulates the diversion of carbon flux towards flavonoid biosynthesis pathway. Silencing and overexpression of CHS encoding gene has resulted in a successful alternation of flavonoids in petunia and tomato, respectively [44], [45]. The silencing and overexpression of *ANR* genes (*ANR1* and *ANR2*) have also redirected the metabolic flux either towards anthocyanins or PAs biosynthesis [20], [46]. The expression of many of genes encoding flavonoid pathway enzymes like CHS, F3H, ANS and ANR is controlled by regulatory genes such as *PAR* (proanthocyanidin regulator). Hence, the overexpression of *CsDFR* and *CsANR* might have affected the expression of selected endogenous genes through influencing the regulators or only the genes encoding endogenous regulatory enzymes of flavonoid pathway showed influence at expression level. Further, *CsDFR* and *CsANR* were continuously expressed by a constitutive promoter 35S and need continuous substrate in transgenic tobacco. This might be avoiding the feedback inhibition of their expression which is otherwise natural possible situation. The upregulation of transcript level of *NtCHS* and *NtANR2* in transgenic tobacco overexpressing *CsDFR* and *CsANR* might further aid to the accumulation of total flavonoids including PAs and monomeric flavan-3-ols.

The accumulation of flavan-3-ols in *CsDFR* and *CsANR* overexpressing transgenic lines was histochemically confirmed by using DMACA staining. The elevation in EC and GC (gallocatechin) content has also been reported earlier in tobacco co-expressing *PAP1* and *MtANR* [19]. However, the EC and GC content in individually overexpressing *MtANR* and *PAP1* tobacco plants was not affected [19]. Our recent study has also documented the elevation in Cat, EC and EGC levels upon *FLS* silencing in tobacco plants. The *FLS* silencing has inhibited the production of flavonols and directed the carbon flux towards biosynthesis of Cat, EC and EGC [31].

Flavan-3-ols act as antioxidant by scavenging free radicals as well as by breaking the free radical chain reactions [5], [38]. High antioxidant potential and free radical scavenging activity of tea has been documented due to its higher levels of polyphenols especially flavan-3-ols [32]. Since *CsDFR* and *CsANR* overexpressing transgenic lines showed higher levels of flavonoids, they were evaluated for their free radical scavenging activity. Both types of transgenics have shown higher total free radical scavenging activities than control tobacco plants. Furthermore, the improved total free radical scavenging activity has encouraged us to check the tolerance of plant against oxidative stress. The overexpression of *CsDFR* and *CsANR* in transgenic lines has lowered H_2_O_2_ mediated cell death, confirming their tolerance against oxidative stress. Similarly, the overexpression of *CsDFR* in transgenic rice has also been reported to provide cell death tolerance [33].

Level of phenolic compounds, especially flavonoids in leaf extracts of various plant species have been documented for plant anti-herbivore defense [37]. Under this category, transgenic tobaccos accumulating caffeine (alkaloids) and rutin (flavonol) have been reported to provide resistance against *S. litura* [34], [47]. Similarly, elevation in the contents of flavan-3-ols and PAs has been reported in response to *Vaccinium myrtillus* infection [48]. The flavonoids have also been reported as insecticidal to *Helicoverpa zea* [4]. In insect gut, these compounds are known to facilitate the conversion of amino acids to more toxic quinones. Such toxic quinones have been reported to reduce the availability of free amino acid and protein by binding to –SH and –NH_2_ groups [4], [39]. There was a strong negative effect on growth and survival of *S. litura* upon feeding on leaves of *CsDFR* and *CsANR* overexpressing transgenic tobacco. Higher levels of monomeric and polymeric flavan-3-ols could be responsible for such an anti-herbivores activity. Their higher content in transgenic tobacco might be acting as anti-nutrient and therefore, reduced the digestibility of protein by *S. litura* either by precipitation of protein or by inhibiting the enzyme activity [4], [5]. These phytochemicals also impart astringency that avoids plant feeding by herbivores [4]–[6]. Thus, higher levels of flavonoids in *CsDFR* and *CsANR* overexpressing transgenic tobacco could be the possible reason for providing protection against feeding by herbivores *S. litura*.

In conclusion, overexpression of tea cDNA *CsDFR* and *CsANR* encoding DFR and ANR enzymatic proteins in transgenic tobacco has induced early flowering and improved seed yield. The *CsDFR*/*CsANR* overexpression has increased the accumulation of flavonoids, thereby improved antioxidant potential and redox state of transgenic tobacco plants. Ultimately, improved antioxidant potential upon *CsDFR* and *CsANR* overexpression in transgenic tobacco has provided the biotic stress tolerance against *S. litura*.

## Supporting Information

Figure S1 **Transcript expression analysis of genes encoding various enzymes of flavonoid biosynthetic pathway in** ***CsDFR*** **and** ***CsANR*** **overexpressing transgenic lines as well as control tobacco plants.**
(TIF)

Figure S2 **HPLC spectra of standard catechin (Cat), epicatechin (EC) and epigallocatechin (EGC) are on the left hand side.** On the right hand side, peaks are identified for Cat, EC and EGC from the extracts of transgenic as well as control tobacco plants by comparing with their respective standard.
(TIF)

Figure S3 **DMACA stained leaf of** ***CsDFR*** **and** ***CsANR*** **overexpressing transgenic tobacco plant vis-à-vis control tobacco plants.**
(TIF)

Figure S4 **HPLC spectra of standard gibberellin (GA_3_) and indole-3-acetic acid (IAA) are on the left hand side.** On the right hand side, peaks for GA_3_ and IAA were identified from the extracts of transgenic as well as control tobacco plants by comparing with their respective standard.
(TIF)

Table S1 **List of primers used for expression analysis of various genes encoding enzymes of flavonoid biosynthetic pathway.**
(TIF)
