# Supplementary figures and images for: Transgenic Tobacco Overexpressing Tea cDNA Encoding Dihydroflavonol 4-Reductase and Anthocyanidin Reductase Induces Early Flowering and Provides Biotic Stress Tolerance
Source: PLoS One. 2013 Jun 18;8(6):e65535. doi: 10.1371/journal.pone.0065535 (PMC3688816; doi:10.1371/journal.pone.0065535)

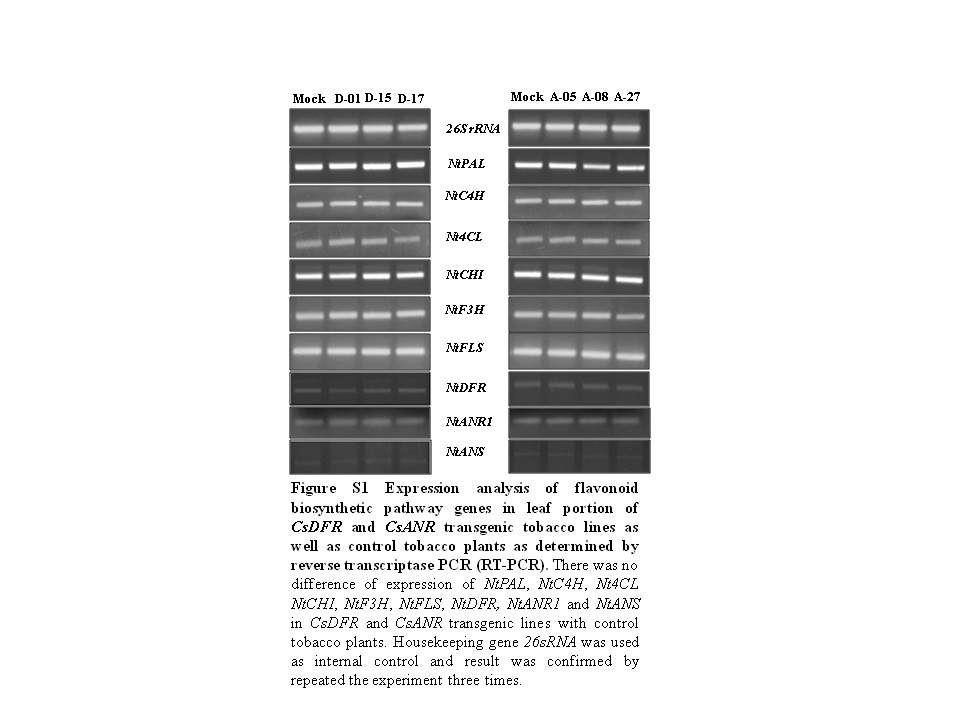

Supplement: Figure S1 — Transcript expression analysis of genes encoding various enzymes of flavonoid biosynthetic pathway in CsDFR and CsANR overexpressing transgenic lines as well as control tobacco plants. (TIF) [file pone.0065535.s001.tif]

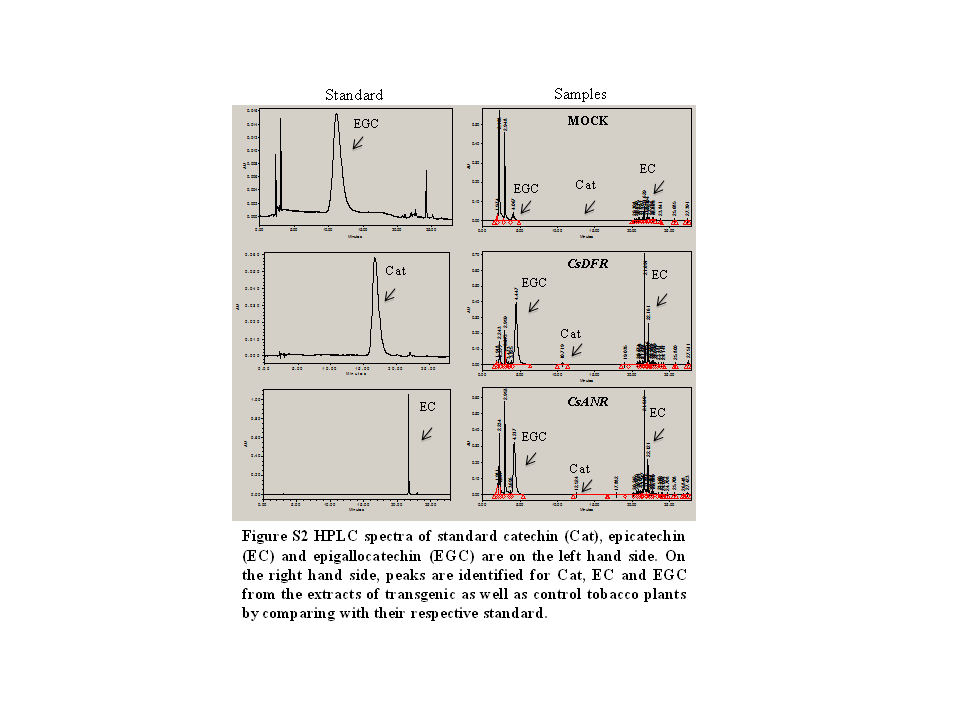

Supplement: Figure S2 — HPLC spectra of standard catechin (Cat), epicatechin (EC) and epigallocatechin (EGC) are on the left hand side. On the right hand side, peaks are identified for Cat, EC and EGC from the extracts of transgenic as well as control tobacco plants by comparing with their respective standard. (TIF) [file pone.0065535.s002.tif]

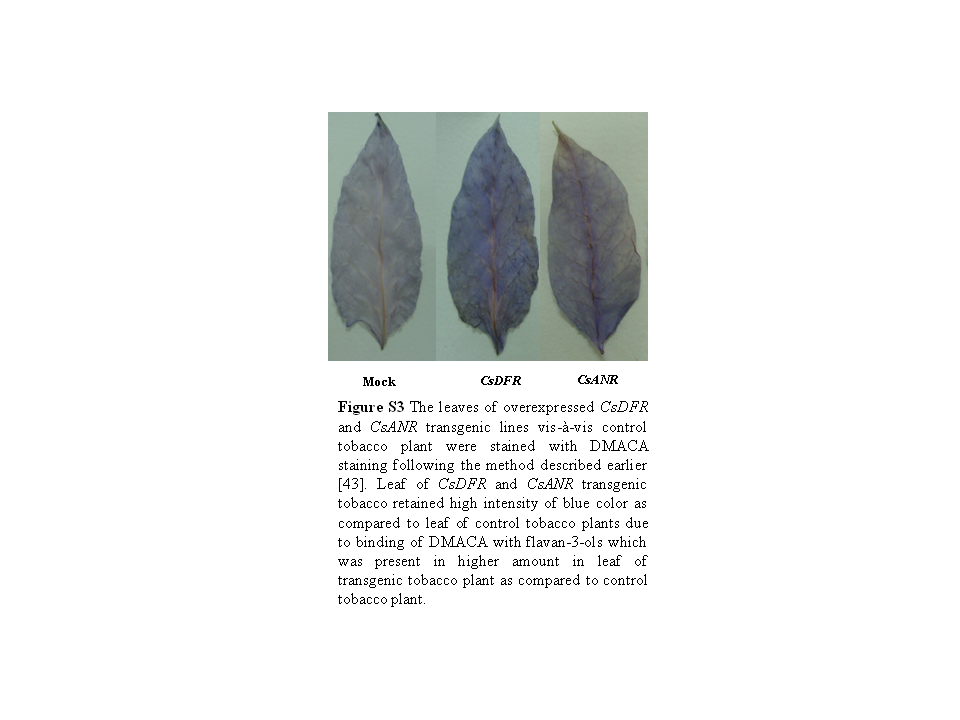

Supplement: Figure S3 — DMACA stained leaf of CsDFR and CsANR overexpressing transgenic tobacco plant vis-à-vis control tobacco plants. (TIF) [file pone.0065535.s003.tif]

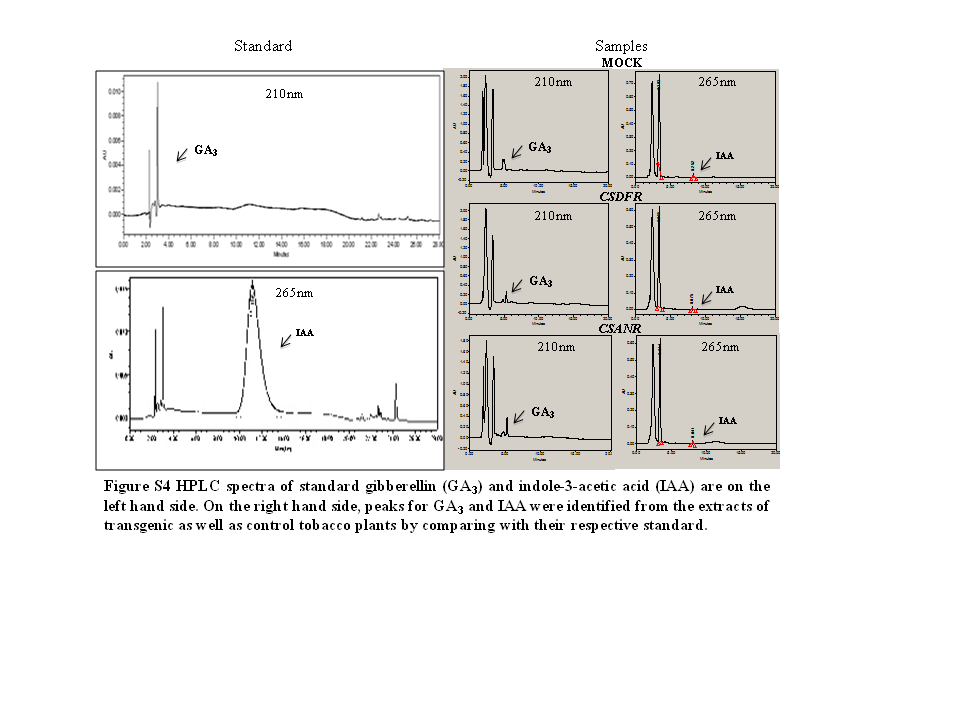

Supplement: Figure S4 — HPLC spectra of standard gibberellin (GA3) and indole-3-acetic acid (IAA) are on the left hand side. On the right hand side, peaks for GA3 and IAA were identified from the extracts of transgenic as well as control tobacco plants by comparing with their respective standard. (TIF) [file pone.0065535.s004.tif]
